# Acute Prostatitis and Septic Shock Following Rectal Spacer Placement: A Case Report of a Pre-brachytherapy Complication

**DOI:** 10.7759/cureus.85099

**Published:** 2025-05-30

**Authors:** Grace E Markey, Puneet Razdan, Suraj Jaipalli, Donald M Rozzell

**Affiliations:** 1 Internal Medicine, Wayne State University School of Medicine, Detroit, USA; 2 Internal Medicine, Henry Ford Health System, Detroit, USA

**Keywords:** brachytherapy, prostate cancer, prostatitis, radiation and clinical oncology, rectal spacer, sepsis, septic shock, severe sepsis

## Abstract

Rectal spacers are commonly used in the treatment of prostate cancer to create a protective barrier to reduce radiation-induced toxicity to the rectum. Despite their safety profile, severe complications such as infections are rare but clinically significant. We present the case of a 69-year-old male with Gleason Grade 3+4 (Score 7) who developed acute prostatitis and septic shock one day after rectal spacer placement. He presented with fever, chills, nausea, and emesis, alongside profound hypotension necessitating vasopressor support. Blood and urine cultures identified *Morganella morganii*, prompting targeted antibiotic therapy with piperacillin-tazobactam. Imaging revealed abdominal edema and mild ascites without abscess or hematoma. The patient recovered with intensive care and was discharged on a prolonged course of antibiotics. This case highlights the rare but severe infectious complications of rectal spacer placement and underscores the importance of early recognition and intervention. Future studies should explore preventive strategies, including prophylactic antibiotics, to mitigate such risks.

## Introduction

A rectal spacer is used to physically separate the rectum from the prostate, reducing radiation exposure and minimizing toxicity during prostate cancer radiotherapy. By increasing the distance between these structures, the spacer decreases the radiation dose to the rectum, which helps decrease treatment-related side effects. Placed within the fascial layer between the rectum and the prostate, the spacer enhances patient safety and treatment tolerance by mitigating the adverse effects of high-dose radiation [1-3].

The hydrogel spacer procedure involves injecting a water-polyethylene glycol mixture to create space between the prostate and rectum, which remains in place for approximately three months and is fully absorbed within six months. Following placement, the patient can undergo external beam radiation therapy, stereotactic body radiotherapy (SBRT), or brachytherapy. Studies have shown that using a rectal spacer before a post-brachytherapy dosimetry scan can minimize rectal radiation exposure, leading to improved treatment outcomes [1-3]. However, this procedure can introduce complications such as infection, particularly acute prostatitis. This condition, usually bacterial in origin, manifests with symptoms like pelvic pain, fever, and urinary issues shortly after spacer placement [1-5].

This case report aims to illuminate the unexpected complications associated with rectal spacer placement in prostate cancer treatment, specifically acute prostatitis and septic shock. While rectal spacers are generally safe, these infections, though rare, can be severe and life-threatening. By presenting the clinical course of a 69-year-old male patient who developed these complications following rectal spacer placement, the report underscores the importance of awareness among healthcare providers regarding the potential risks of rectal spacers. The unique learning value of this case lies in its emphasis on the need for early recognition and management of such adverse events, particularly in the context of radiation oncology. This report highlights the necessity for diligent monitoring and prompt intervention to mitigate complications, ultimately contributing to improved patient safety and outcomes in prostate cancer therapies.

## Case presentation

A 69-year-old male with a history of prostate cancer, Gleason Grade 3+4 (Score 7), presented to the emergency department one day after undergoing rectal spacer placement as part of pre-brachytherapy preparation. He reported experiencing fever, chills, nausea, non-bloody vomiting, and mild diffuse abdominal pain.

On initial evaluation, the patient was febrile with a maximum temperature of 101.6°F, tachycardic with a heart rate of 98 bpm, and hypotensive with a blood pressure of 83/59 mmHg (mean arterial pressure (MAP) 67). Oxygen saturation was 100% on room air. He denied symptoms such as chest pain, shortness of breath, urinary abnormalities, or bowel irregularities.

The patient received intravenous fluid resuscitation upon admission. However, his condition worsened, with a further decline in systolic blood pressure into the 60s. A rapid response was initiated, and norepinephrine was started. He was subsequently transferred to the medical intensive care unit (MICU) for management of suspected septic shock. Laboratory studies revealed elevated lactate, leukocytosis, and acute kidney injury (Table 1). Blood and urine cultures later identified *Morganella morganii* as the causative organism. Antibiotic therapy with piperacillin-tazobactam was promptly initiated following consultation with infectious disease specialists.

**Table 1:** Laboratory findings in a patient with septic shock prior to antibiotics showing elevated WBC, AST, ALT, and lactic acid, along with decreased RBC, Hgb, and platelet count, consistent with systemic inflammatory response and multi-organ involvement.

Lab Test	Patient Data	Reference Range	Units
White Blood Cell (WBC) Count	17.95 (H)	4.0-11.0	K/µL
Red Blood Cell (RBC) Count	3.35 (L)	4.7-6.1 (male), 4.2-5.4 (female)	million/µL
Hemoglobin (Hgb)	10.6 (L)	13.5-17.5 (male), 12.0-15.5 (female)	g/dL
Platelet (PLT) Count	49 (L)	150-450	K/µL
Aspartate Aminotransferase (AST)	61 (H)	10-40	IU/L
Alanine Aminotransferase (ALT)	86 (H)	7-56	IU/L
Lactic Acid	4.0 (H)	0.5-2.0	mmol/L

**Figure 1:**
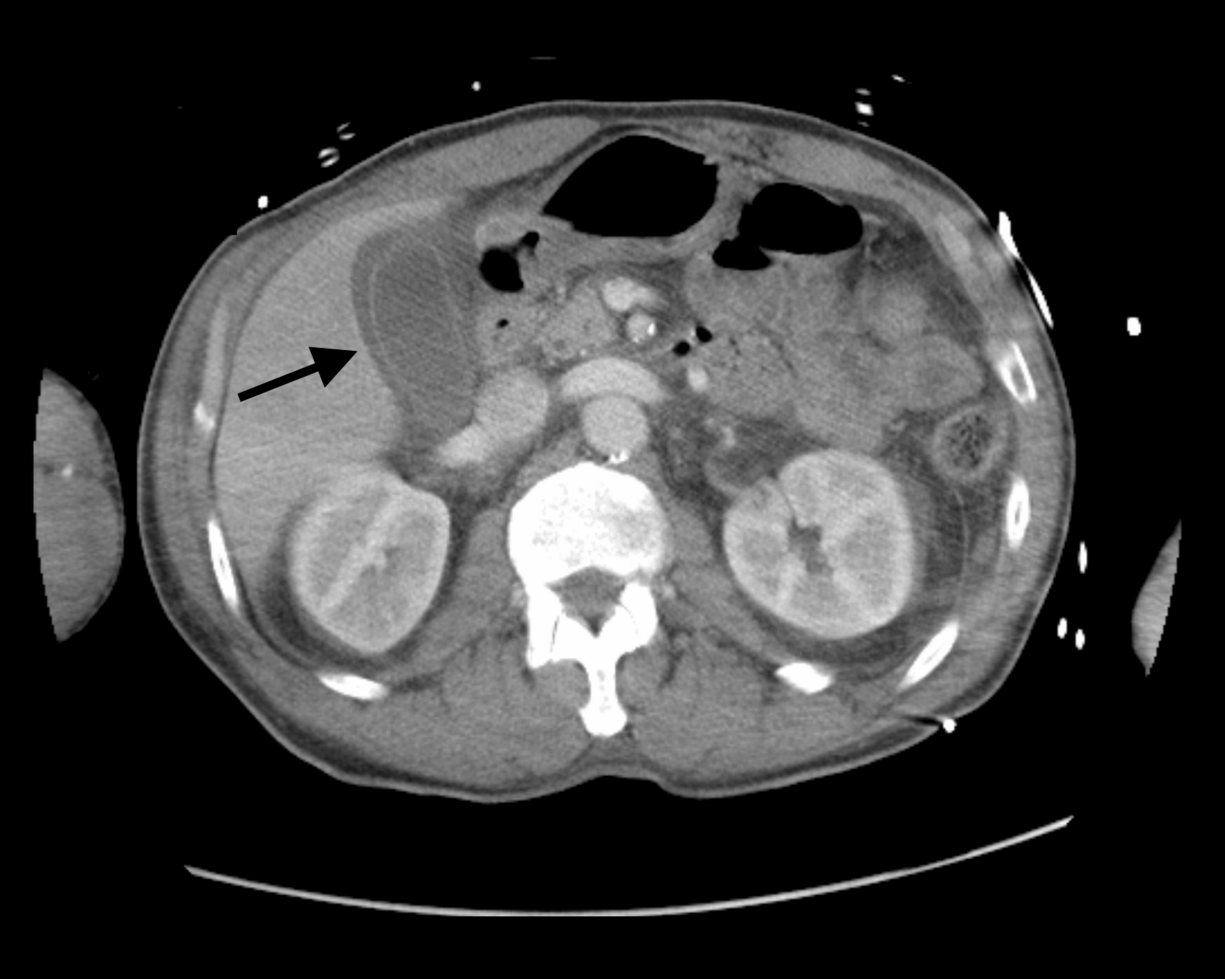
Axial CT scan of the abdomen and pelvis demonstrating abdominal edema, mild ascites, and trace bilateral pleural effusions without evidence of abscess, hematoma, or hydronephrosis (black arrow).

**Figure 2:**
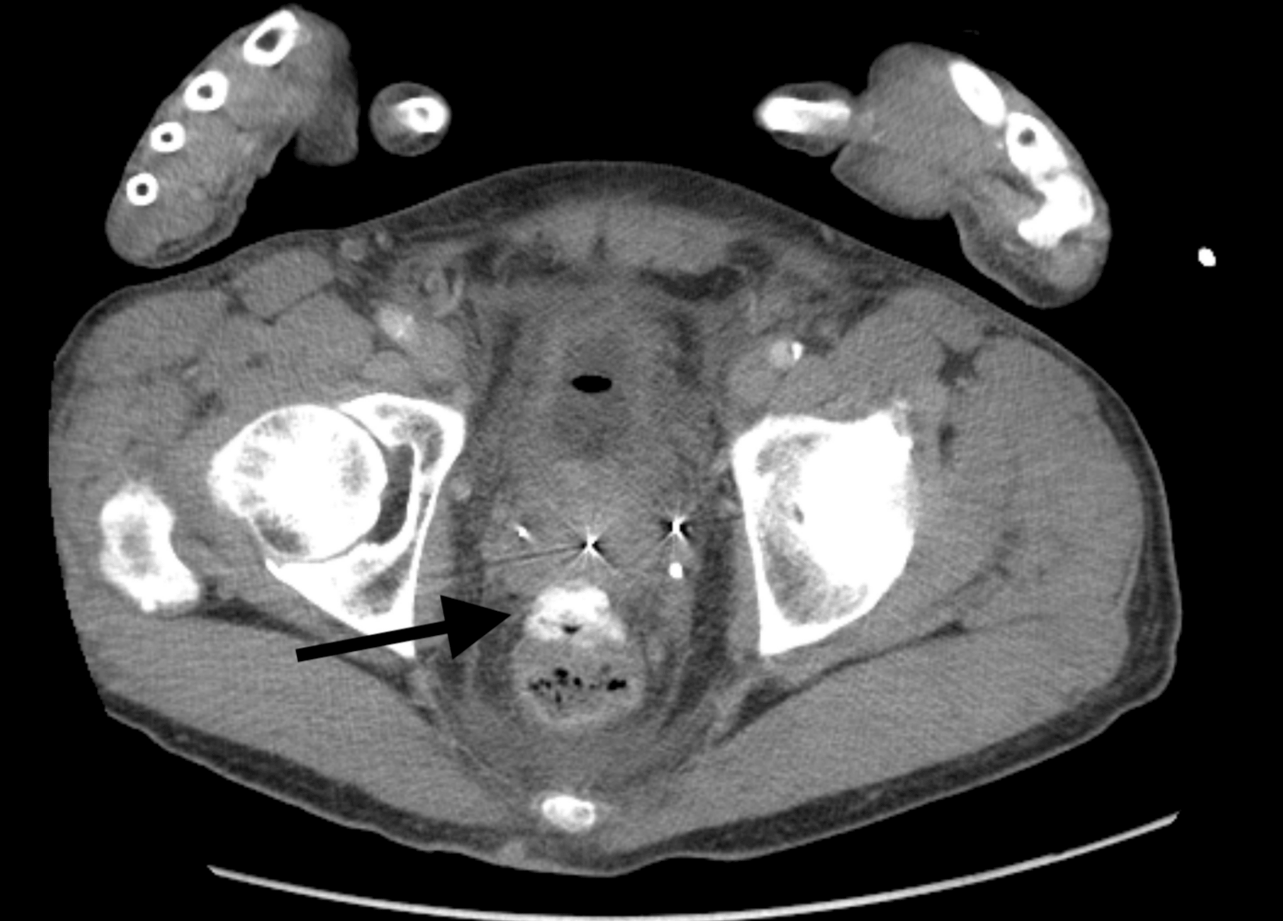
Axial CT image at the level of the prostate demonstrating a fluid collection with features suggestive of a possible abscess (black arrow).

Computed tomography (CT) imaging revealed abdominal edema, mild ascites, trace bilateral pleural effusions, a fluid collection with features suggestive of a possible abscess, and no evidence of hematoma or hydronephrosis (Figures 1-2). A Foley catheter was placed for urinary drainage, aiding in the management of acute kidney injury. Over the next few days, the patient showed clinical improvement, allowing us to wean off norepinephrine and transition to oral midodrine for continued blood pressure support. He was eventually transferred to the medical floor in stable condition and continued on intravenous antibiotics.

The patient was discharged with a four-week course of sulfamethoxazole-trimethoprim and instructed to follow up with infectious disease and urology specialists. During his hospital stay, painful oral ulcers indicative of a Herpes simplex virus type 1 (HSV-1) flare-up were noted and successfully managed with valacyclovir.

## Discussion

This case highlights the rare yet serious complication of acute prostatitis and septic shock following rectal spacer placement. While rectal spacers significantly reduce the radiation dose to the rectum by minimizing the volume exposed to high-dose radiation, their placement can introduce infection, potentially leading to severe outcomes. The occurrence of *M. morganii* as the causative pathogen in this patient's septic presentation underscores the need for heightened awareness of potential infectious complications post-procedure. Symptoms such as fever, hypotension, and nausea should prompt immediate medical attention in patients following rectal spacer placement. Existing literature has documented various adverse events associated with rectal spacers, but instances of acute prostatitis leading to severe systemic infection are infrequently reported. This case contributes to the understanding of such rare complications, emphasizing the importance of early recognition and management to prevent severe outcomes. Recognizing these symptoms early can be critical in preventing complications, making it essential for healthcare providers to remain vigilant in monitoring patients after the procedure.

Spacer technology serves as an effective method to minimize radiation dose to the anterior rectal wall, significantly enhancing the safety profile of brachytherapy. While the advantages of dose sparing are considerable, it is crucial to weigh these benefits against the low but potential risks of complications, including rectal perforation and other adverse events. A balanced understanding of these factors is essential for clinicians, as it informs treatment planning and patient counseling, ensuring that the decision to use spacers is made with a comprehensive view of both their efficacy and associated risk [6].

In reviewing the existing literature on post-rectal spacer complications, various adverse events associated with Space Organs At Risk (SpaceOAR) placement prior to brachytherapy are documented. Complications such as rectal wall infiltration may increase radiation-induced injuries, while rare but severe cases of rectal perforation can delay radiation therapy due to symptomatic concerns [7-10]. Other risks include gel extension to the base of the penis, raising the possibility of rectourethral fistula formation, and persistent nodular formations where the gel can mimic disease recurrence on imaging. Intravasation of the gel into the periprostatic venous complex, though typically clinically insignificant, requires careful interpretation to ensure adequate gel volume for rectal protection during therapy [8,9]. Severe complications reported in the literature include acute pulmonary embolism, prostatic abscess, severe anaphylaxis, and infections that may require bowel or urinary diversion [9]. Additionally, recent reviews of post-market SpaceOAR data from the Manufacturer and User Facility Device Experience (MAUDE) database highlight the importance of vigilant monitoring and management to improve patient outcomes, given reports of infections, rectal ulcerations, and rare fatal complications [10].

Early recognition and management of acute bacterial prostatitis are essential due to the rapid onset of symptoms and the risk of serious complications. Acute bacterial prostatitis, a bacterial infection of the prostate, can cause sudden urinary symptoms, such as painful urination, increased frequency, and urgency, as well as pelvic pain and systemic signs like fever, chills, and a general feeling of unwellness [11]. Patients experiencing these symptoms after a recent urological procedure should promptly seek medical care, as delays in treatment can lead to increased morbidity and even mortality. Laboratory tests, including urinalysis and urine cultures, help confirm the diagnosis, while blood cultures are necessary if fever surpasses 101.1°F or if sepsis is suspected.

Educating patients about post-procedural infection risks and preventive strategies, such as using antibiotics before certain procedures, can be instrumental in reducing infection. However, patients should be aware that antibiotic resistance can limit effectiveness. Following any prostate-related procedure, patients should monitor for early signs of infection and seek medical care immediately if symptoms arise.

Future studies should explore the role of targeted prophylactic antibiotic regimens, particularly in patients at high risk of infection. For example, patients with conditions that predispose them to severe infections, such as those with artificial heart valves or a history of endocarditis, already receive antibiotic prophylaxis before certain medical procedures. Similarly, prophylactic antibiotics before rectal spacer placement may be beneficial for patients with elevated infection risk due to underlying medical conditions or immunosuppression. Additionally, future strategies should emphasize improving patient education regarding post-operative signs of infection, enhancing monitoring protocols, and refining management approaches to mitigate the risk of similar complications.

## Conclusions

This case underscores the importance of recognizing and managing severe complications associated with rectal spacer placement in prostate cancer therapy. The rare presentation of acute prostatitis and septic shock reinforces the need for heightened vigilance, particularly in patients presenting with early signs of infection. Timely intervention and multidisciplinary collaboration are crucial in such cases to optimize patient outcomes. Clinicians should remain aware of these potential risks, and current clinical guidelines regarding prophylactic antibiotic use in this setting should be further examined, as gaps in prevention and management may exist. Enhanced awareness and proactive care can help mitigate complications and improve patient safety in radiation oncology.
